# MicroRNA-199a Inhibits Cellular Autophagy and Downregulates IFN-β Expression by Targeting TBK1 in *Mycobacterium bovis* Infected Cells

**DOI:** 10.3389/fcimb.2018.00238

**Published:** 2018-07-10

**Authors:** Jie Wang, Tariq Hussain, Ruichao Yue, Yi Liao, Qiang Li, Jiao Yao, Yinjuan Song, Xin Sun, Nan Wang, Lei Xu, Srinand Sreevatsan, Deming Zhao, Xiangmei Zhou

**Affiliations:** ^1^State Key Laboratories for Agrobiotechnology, Key Laboratory of Animal Epidemiology and Zoonosis, Ministry of Agriculture, National Animal Transmissible Spongiform Encephalopathy Laboratory, College of Veterinary Medicine, China Agricultural University, Beijing, China; ^2^China Institute of Veterinary Drug Control, Beijing, China; ^3^Pathobiology and Diagnostic Investigation, Michigan State University, East Lansing, MI, United States

**Keywords:** *M. bovis*, immunity, autophagy, IFN-β, microRNA, TBK1

## Abstract

The mechanism by which microRNAs (miRNAs) modulate innate immunity and autophagy has not been fully elucidated in *Mycobacterium bovis* (*M. bovis*) infections. In this study, we identified that miR-199a inhibited key innate immune responses and autophagy in murine macrophages infected with *M. bovis*. Using *ex vivo* and *in vitro* approaches we show that the expression of miR-199a was significantly increased during *M. bovis* infection. Furthermore, miR-199a suppressed autophagy and interferon-β (IFN-β) production by directly targeting TANK-binding kinase 1 (TBK1) mRNA in both J774a.1 and BMDM cells. Upregulation of miR-199a or TBK1 silencing (siTBK1) inhibited maturation of autophagosomes and increased *M. bovis* survival. Our results demonstrate that, by targeting of TBK1, miR-199a modulates innate immune responses and promote the intracellular survival and growth of *M. bovis*.

## Introduction

*Mycobacterium bovis* is a causative agent of tuberculosis in multiple species of animals, including cattle. It can also cause disease in humans with lesions undistinguishable from those caused by *M. tuberculosis*. It is estimated that more than 50 million cattle in the world are infected with *M. bovis*, resulting in annual economic losses of about $ 3 billion (Fend et al., 2005). Macrophages offer the first line of anti-*Mycobacterium* immune defense via various pathogen recognition receptors. Thus, a better understanding of the interaction between *M. bovis* and macrophages would contribute to the better control of bovine tuberculosis. Non-coding RNA plays a very crucial role in this process. Micro-RNAs (miRNAs) are non-coding single-stranded RNAs with a length of about 22 nucleotides, which regulates gene expression by initiating degradation of mRNAs or by inhibiting protein translation (Ambros et al., 2003; Friedman et al., 2009). Recent research has demonstrated that miRNAs have an important role in immune regulation in infectious and some autoimmune diseases (Desvignes et al., 2012; Khan et al., 2013). Several other studies have clearly established the role of miRNAs in regulation of the immune response during *M. tuberculosis* infection (Maudet et al., 2014; Mehta and Baltimore, 2016). In addition, it is reported that inflammatory signaling pathways are likely regulated by mycobacteria through differential expression of miRNAs (Junger, 2011; Ma et al., 2011).

TANK-binding kinase 1 (TBK1) plays a key role in innate immune responses, such as transcription and autophagy, to virulent *M. tuberculosis* (Stanley et al., 2003; Watson et al., 2012). It is a ubiquitously expressed serine/threonine-protein kinase, a member of the IkappaB kinase family, which is encoded in the *Tbk1* gene (Yu et al., 2012). TBK1 has the ability to affect the secretion of inflammatory mediators such as IFN-β, IL-6, and TNF-α (Perry et al., 2004; Marchlik et al., 2010; Xie et al., 2012). One of its key functions is to mediate type I interferons by the induction of cytosolic double-stranded DNA (Saitoh et al., 2009). TBK1 is also involved in selective autophagy pathways for efficient clearance of pathogens, protein aggregates, and mitochondria (Wild et al., 2011; Pilli et al., 2012; Korac et al., 2013; Matsumoto et al., 2015). Autophagy is a barrier against intruding *M. tuberculosis*. TBK1 is recruited and activated through NDP52 and OPTN to ubiquitin-decorated intracellular bacteria. It then enhances LC3 combining affinity and autophagic removal of cytosolic Salmonella (Thurston et al., 2009; Wild et al., 2011). Hence, TBK1 mediates the crosstalk between autophagy and the innate immune response but the possible role of miRNAs in regulating TBK1 function during *M. bovis* infection has not been sufficiently studied.

It is known that miR-199b is downregulated during early stages of infection with *M. tuberculosis*, although the authors studied its expression only up to 24 h in mice model of infection (Kumar et al., 2015). There is only single nucleotide difference between miR-199a and miR-199b while similarly target TBK1 including other predicted targets (Supplementary Figure S1). Since *M bovis* have high homology with *M tuberculosis* and can cause infection in humans and others species of animals. Therefore, we hypothesized that miR-199a may be a regulator that modulates immune responses associated with *M. bovis* infection. We found that the expression of miR-199a was initially downregulated, but increased after 24 h in response to *M. bovis* infection in murine macrophages. Early studies investigate various innate immune signaling pathways in murine macrophages infected with *M. bovis*. Previous study reported that stimulation of murine peritoneal macrophages and bovine alveolar macrophages with IFN-γ and LPS, similarly induced growth inhibition of virulent *M. bovis* (Aldwell et al., 2001). Another study, demonstrated that IFN-β release increases in murine macrophages exposed to *M. bovis* and this requires the activation of the DNA sensor of interferon-γ inducible protein 204 (IFI204) (Liu et al., 2017). Consistent with previous studies we selected murine macrophages in our current study to evaluate the role of miR-199a in *M. bovis* infection. We observed a time-dependent upregulation of miR-199a expression in response to *M. bovis* infection in macrophages. In addition, exogenous miR-199a significantly inhibited cellular autophagy, suppressed the induction of IFN-β and led to a higher bacterial burden in infected macrophages. Furthermore, we investigated the inhibitory role of miR-199a in the regulation of cellular autophagy is mediated by targeting TBK1. In the current study, we evaluated the regulation of miR-199a in mouse macrophages in response to *M. bovis* infection and demonstrated its possible functionality in the innate immune response. This study provides a potential new anti-tubercular therapeutic modality to enhance clearance of intracellular mycobacteria by modulation of miR-199a expression.

## Materials and methods

### Cell cultures

J774a.1 cells and the HEK 293 cells were obtained from the Cell Culture Center, Xiehe Medical University (Beijing, China). The cells were cultured in a humidified incubator at 37°C with 5% CO2 in DMEM (Hyclone, Logan, UT, USA) supplemented with 10% FBS (Gibco, Grand Island, NY, USA), 100 μg/ml streptomycin and 100 U/ml penicillin (Gibco). Mouse bone marrow monocyte-derived macrophages (BMDMs) were isolated from the femurs of 6 to 8-weeks-old female C57BL/6 mice, and cultured in six-well plates (Corning) for 7 days in RPMI1640 (Hyclone) supplemented with 10 ng/ml M-CSF (Pepro Tech), 10% FBS, 100 microgram/ml streptomycin and 100 U/ml penicillin (Gibco).

### Bacterial culture

Virulent *M. bovis* Beijing strain number C68004 was provided by the China Institute of Veterinary Drug Control (CVCC, China), and grown in 7H9 Middlebrook media (Difco) supplemented with 10% albumin-dextrose-catalase (ADC) enrichment solution, 2 g/l sodium pyruvate and 0.05% Tween-80 at 37°C under biosafety conditions level 3 (BSL3).

### Colony-forming units (CFU) assay

To assess bacterial viability, BMDM and J774a.1 (2 × 10^5^cells in each well) were cultured in 12-well plates overnight and infected with *M. bovis* (MOI 10:1) strains for 3 h and then washed thrice with warm PBS to remove extracellular bacteria in BSL3 safety conditions. Thereafter, the infected cells were incubated for the indicated time periods and lysed at the specified time points with 0.1% Triton X 100. Quantitative culturing was performed using 10-fold serial dilutions. Aliquots of each dilution were inoculated in triplicate on Middlebrook 7H11 agar plates supplemented with 10% ADC enrichment solution and 0.05% Tween-80. After incubation for 2 weeks, colonies on plates were counted. The survival rate was calculated by comparing to the control.

### Mice and *M. bovis* infection

C57BL/6 mice were obtained from Vital River Laboratories (Beijing, China) and accommodated in the biosafety level 3 laboratory facilities of China Agricultural University. Mice were divided into two groups: one group was intranasally infected with *M. bovis* (at a dose of 200 CFU/mouse) and a second group was intranasally treated with Phosphate Buffered Saline (PBS) (*n* = 5). After 4 weeks, mice were sacrificed and lungs, spleens, and livers were harvested for further analysis. All animal work was approved by the Laboratory Animal Ethical Committee of China Agricultural University (Protocol 20110611-01).

### Cell transfection

Cells were allowed to attach overnight in 12-well plates (2 × 10^5^ cells in each well). The following day, J774a.1 cells and BMDMs were transfected with 50 nM control mimic, control inhibitor, miR-199a mimic or miR-199a inhibitor (GenePharma, Shanghai, China) using Lipofectamine 2000 (Invitrogen, CA). For TBK1 knockdowns, cells were transfected with 10 nM SMART pool reagents (Dharmacon, Waltham, MA). Control mimic (control) was used as a control for siTBK1 or miR-199a mimic. The sequence of control mimic, control inhibitor, miR-199a mimic, miR-199a inhibitor, control, siTBK1 are shown in Supplementary Table S1. After 6 h, the original medium was replaced with fresh medium supplemented with 10% FBS. Forty-eight hours post-transfection, macrophages were infected at a multiplicity of infection (MOI) of 10 with *M. bovis* in cell culture medium without antibiotic. Following incubation for 3 h at 37°C in 5% CO_2_, the supernatant was discarded and each well was washed three times with sterile phosphate-buffered saline (PBS) to remove non-adherent *M. bovis*. After washing, fresh DMEM (J774a.1) and RPMI 1640 (BMDM) medium supplemented with 10% FBS was added for the specified time period. For autophagy analyses, J774a.1 cells were infected with adenovirus expressing mCherry-GFP-LC3B fusion protein (Ad-mCherry-GFP-LC3B) (Beyotime Beijing, China) at a multiplicity of infection (MOI) of 10 for 24 h. For blockade of type I interferon signaling, J774a.1 cells and BMDMs were stimulated at 37°C for various periods of time with *M. bovis* at a MOI of 10 with or without prior incubation with 1 μg/ml of an IFNAR1 blocking antibody (clone MAR1-5A3; Biolegend) for 2 h at 37°C.

### Quantitative real-time PCR

Total RNA (including miRNA) was extracted using Trizol Reagent (Invitrogen), and the concentration and integrity were detected by NanoDrop 2000 machine (Thermo Scientific, Waltham, MA, USA). Reverse transcription of 100 ng RNA was performed by the Revert Aid first-strand cDNA synthesis Kit (Fermentas, Glen Burnie, MD, USA) according to the manufacturer's instructions, and the integrity and concentration of cDNA were detected by NanoDrop 2000 machine to confirm same amount of cDNA used for different samples. AceQ qPCR SYBR Green Master Mix kit (Vazyme Biotech, Nanjing, China) was used for the amplifcation of mRNA's genes, by using the 700 Fast Real-Time PCR Systems (ViiA7 Real-time PCR, ABI). All the primers used in the present study are shown in Table 1. Standard PCR cycle parameters were as follows: 95°C for 300 s, followed by 40 cycles of 95°C for 10 s, 60°C for 30 s. For miR-199a detection, reverse transcription of 100 ng RNA was done by using miRcute Plus miRNA First strand cDNA synthesis kits (TIANGEN BIOTECH Beijing, China) according to the manufacturer's instructions, and the integrity and concentration of cDNA were detected by NanoDrop 2000 machine to confirm same amount of cDNA used for different samples. Quantitative real-Time PCR was performed by using miRcute miRNA qPCR Detection Kit (Tiangen Biotech, Beijing, China) A universal miR qPCR primer (possessing the binding site with universal adaptor primer, included in the kit), miRNA-199a primer, and U6 primer as internal reference (sequence complementary to the miRNA) were used to complete the real-time PCR reaction. All reactions were performed in triplicate. miRNA qRT-PCR cycle parameters were as follows: 94°C for 2 min, followed by 45 cycles at 94°C for 20 s, and 60°C for 34 s. The relative expression levels of miRNAs and mRNAs were determined by a comparative Ct (ΔΔCt) method.

**Table 1:** Primers used for Quantitative Real-Time PCR.

**Gene name**	**Forward primer (5′-3′)**	**Reverse primer (5′-3′)**
IFN-β	AAGAGTTACACTGCCTTTGCCAT	CACTGTCTGCTGGTGGAGTTCATC
TBK1	TTACAAGAAACTCTGCCTCA	TAACCACGCCTTCCATCT
GAPDH	CGACTTCAACAGCAACTCCCACTCTC	TGGGTGGTCCAGGGTTTCTTACTCCTT

### Nuclear and cytoplasmic protein extraction

NE-PER Nuclear and Cytoplasmic Extraction Reagents was obtained from Thermo Scientific, protein was extracted from nuclei and cytoplasms as per manufacturers' instructions.

### Western blot

Total protein was isolated using RIPA Lysis Buffer (Beyotime). Equal amounts of protein were separated by SDS-PAGE electrophoresis, transferred onto PVDF membranes (Millipore Corporation, Billerica, MA) and probed with rabbit monoclonal anti-TBK1/NAK (D1B4) antibody (3504), rabbit monoclonal anti-phospho-TBK1/NAK (Ser172) (D52C2) antibody (5483), rabbit monoclonal anti-IRF-3 (D83B9) antibody (4302), rabbit monoclonal anti-phospho-IRF-3 (Ser396) (D6O1M) antibody (29047) (Cell Signaling Technology, Boston, Mass, USA), and mouse anti-Map-LC3 antibody (sc-376404) (Santa Cruz, CA, USA). This was followed by HRP-labeled secondary antibody (Protein Tech, Wuhan, Hubei, China). Bands were developed with ECL substrate, visualized using BIO-RAD imaging system and analyzed by Image J analysis software.

### Plasmid constructs and luciferase assay

The binding elements for miR-199a at the *Tbk1* 3′UTR mRNAs were obtained by PCR amplification using mouse genomic DNA as template and cloned into pMir-Reporter Luciferase vector (GenePharma). A mutant form of *Tbk1* 3′UTR was cloned into a pmirGLO vector by site-directed mutagenesis using a WT clone. HEK293, J774a.1, and BMDMs were cultured in 24-well plates (2.5 × 10^4^ cells/well) overnight and transfected with 50 nM control mimics or 50 nM miR-199a mimics using a Lipofectamine 2000. Six hours post-transfection, cells were again transfected with 100 ng pMir-Reporter constructs of *Tbk1* wild- and mutant type of plasmid. Twenty-four hours after transfection, cells were lysed in lysis buffer, and a luciferase assay was performed using the Dual Luciferase reporter system (Promega), according to the manufacturer's instructions by using Orion II Microplate Illuminometer (Titertek-Berthold, South San Francisco, CA, USA). Firefly luciferase units were normalized against Renilla luciferase units to control for transfection efficiency. Relative activities were expressed as the fold change in luciferase activity. The results were obtained from three independent experiments, and all samples were collected in three replicates in each experiment.

### Immunofluorescence assay

*M. bovis* staining as described previously (Chunfa et al., 2017). Briefly, 10 μl of a log-phase *M. bovis* culture were transferred into a 15 ml conical tube. Mycobacterial cultures were centrifuged at 3,000 rpm for 5 min, the supernatant fraction was carefully removed and the pellet washed twice with 10 ml of 1x PBS. This was centrifuged at 3,000 rpm for 5 min and the supernatant fraction removed and discarded. The pellet was resuspended in 1 ml of PBS and the cell suspension transferred into a 1.5 ml microcentrifuge tube. Ten microliters of Alexa 488 caboxylic acid succinimidyl ester stock solution was added to the tube to make a final concentration of 10 mg/ml. The tube was wrapped with foil and incubated at 37°C for 45–60 min on a shaker. Mycobacteria were pelleted by centrifugation at 10,000 rpm for 3 min at room temperature. The supernatant was removed and washed twice with 1 ml of PBS. The mycobacterial pellet was resuspended in 6 ml of complete DMEM. Immunofluorescence was performed as described previously (Chunfa et al., 2017). Briefly, cells were fixed in 4% paraformaldehyde for 10 min. Non-specific binding sites were blocked in Blocking Buffer for Immunol Staining (Beyotime) for 1 h. Cells were then incubated for 1 h with mouse anti-Map-LC3 antibody (sc-376404) (Santa Cruz, CA, USA) or rabbit monoclonal anti-IRF3 (D83B9) antibody (4302) (Cell Signaling Technology), followed by *M. bovis* staining. Washed three times in PBS, cells were then incubated for 1 h with the specific Alexa Fluor- conjugated secondary antibodies (Cell Signaling Technology). Nuclei were stained with DAPI (Sigma). Cells were examined immediately with an OLYMPUS microscope.

### Transmission electron microscopy

Transmission electron microscopy (TEM) was used to detect autophagosomes as previously described (Chunfa et al., 2017). J774a.1 macrophages were transfected with control, miR-199a mimic or siTBK1 for 24 h, and then infected with *M. bovis* for 24 h. Cells were fixed in 2.5% v/v glutaraldehyde in 0.2 M phosphate buffer. Fixed cells were post-fixed in 2% osmium tetroxide and 100 mM cacodylate buffer, dehydrated with increasing concentrations of ethanol and gradually infiltrated with Epon resin (Pelco, Redding, CA). Thin sections were stained with uranyl acetate and lead citrate and examined using transmission electron microscope.

### Enzyme-linked immunosorbent assay (ELISA) assay

The level of IFN-β was assessed from the cell culture supernatants using a mouse interferon-β ELISA kit (Cusabio, Wuhan, Hubei, China) following manufacturer's instructions.

### Statistical analysis

Statistical analyses were performed using two-tailed Student's *t*-test. Comparisons between groups were performed using ANOVA (Prism GraphPad 5 Software). All data are presented as mean ± *SD* of three independent experiments. Significant differences were assigned at *P* < 0.05, < 0.01, and < 0.001, denoted by *, **, and ***, respectively.

### Animal ethics statement

All protocols and procedures were performed according to the Chinese Regulations of Laboratory Animals—The Guidelines for the Care of Laboratory Animals (Ministry of Science and Technology of People's Republic of China) and Laboratory Animal Requirements of Environment and Housing Facilities (GB 14925–2010, National Laboratory Animal Standardization Technical Committee). The license number associated with their research protocol was 20110611–01 and the animal study proposal was approved by The Laboratory Animal Ethical Committee of China Agricultural University.

## Results

### *M. bovis* infection markedly increases miR-199a expression in macrophages *in vitro* and *in vivo*

**Figure 1:**
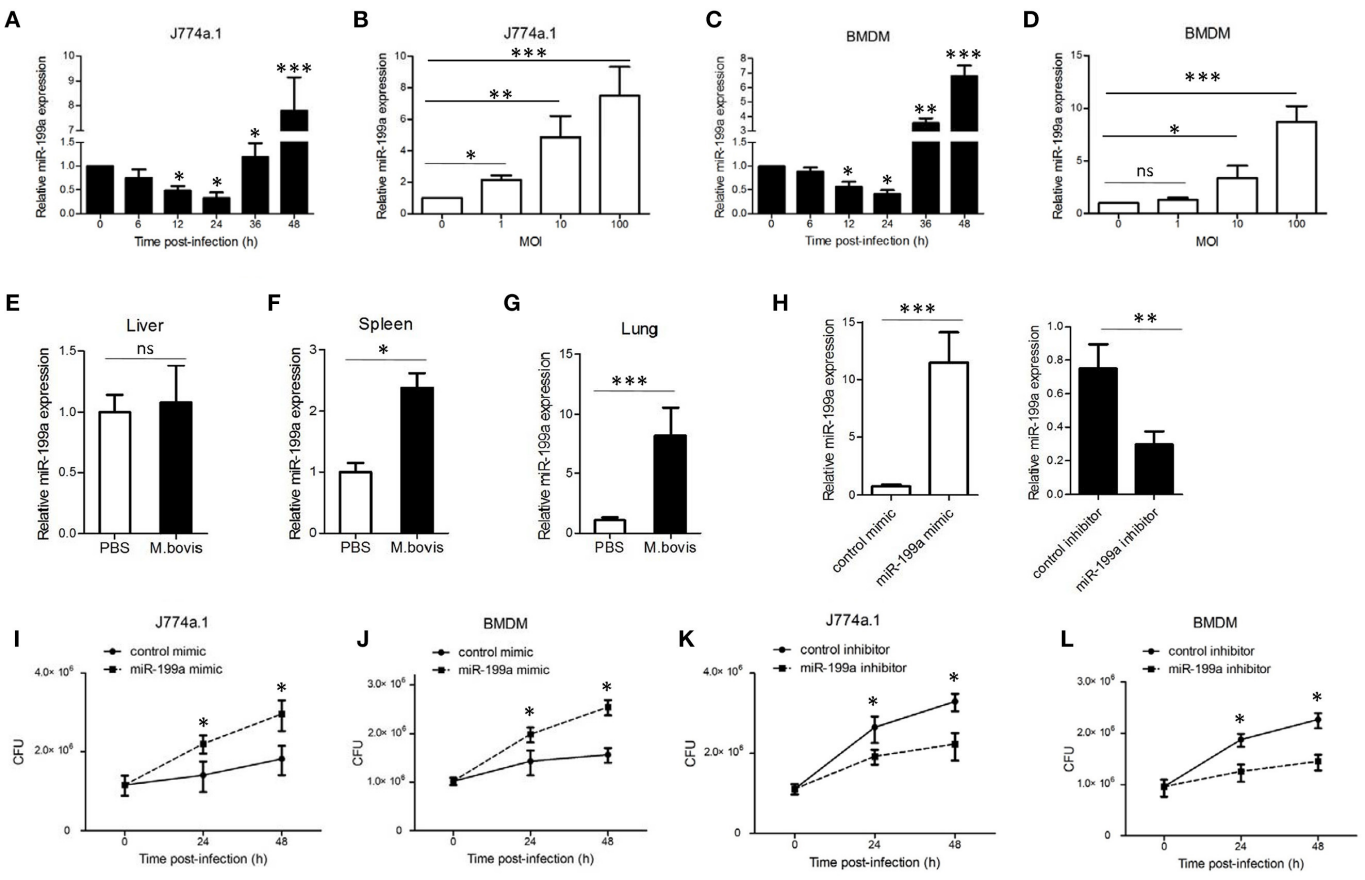
*Mycobacterium bovis* (*M. bovis*)-induced upregulation of miR-199a is associated with increased bacterial survival. miR-199a expression was detected by Quantitative Real-Time PCR(qRT-PCR). **(A,B)** J774a.1 cells or **(C,D)** mouse bone marrow monocyte-derived macrophages (BMDMs) were infected with *M. bovis* at a multiplicity of infection (MOI) of 10 for the indicated time or for the indicated MOI for 48 h. **(E–G)** Mice (*n* = 5) were intranasally infected with *M. bovis* (at a dose of 200 CFU/mouse) or PBS. After 4 weeks, mice were sacrificed and livers, spleens, and lungs were collected for qRT-PCR. **(H)** Cells were transfected with miR-199a mimic or inhibitor for 24 h and then infected with *M. bovis* at a MOI of 10 for 24 h. miR-199a expression was measured by qRT-PCR. Forced expression **(I,J)** or inhibition **(K,L)** of miR-199a enhanced or reduced bacterial survival at a MOI of 10 for 24 and 48 h post-infection. Data is presented as mean ± *SD* in three independent experiments (*P < 0.05; **P < 0.01; ***P < 0.001).

To explore the expression of miR-199a during *M. bovis* infection, J774a.1 cells and mouse bone marrow monocyte-derived macrophages (BMDMs) were infected with *M. bovis*. Quantitative Real-Time PCR (qRT-PCR) showed that the expression of miR-199a in *M. bovis*-infected cells gradually decreased in the first 24 h, but markedly increased at 36 h and 48 h (Figures 1A,C). Figures 1B,D show that *M. bovis* infected cells responded by gradually increasing expression of miR-199a in a multiplicity of infection (MOI)-dependent manner at 48 h. In addition, the expression of miR-199a was highly upregulated in spleens and lungs (Figures 1F,G) from C57BL/6 mice challenged with *M. bovis* for 4 weeks, although the change of miR-199a expression in the liver was not significant (Figure 1E). These results clearly indicate that *M. bovis* infection induces miR-199a expression. This apparent association of miR-199a upregulation with *M. bovis* infection prompted us to investigate further the role of miR-199a in *M. bovis* infection.

### miR-199a promotes *M. bovis* survival in macrophages

qRT-PCR results indicated that transfection with miR-199a mimic markedly increased miR-199a expression, whereas transfection with miR-199a inhibitor significantly decreased miR-199a expression in cells during *M. bovis* infection (Figure 1H). In order to survey the role of miR-199a in bacterial survival, we directly scored for bacterial CFU in infected cells after transfection with either miR-199a mimic or miR-199a inhibitor. Survival of *M. bovis* increased in the presence of miR-199a mimic (Figures 1I,J). Conversely, survival of *M. bovis* was compromised in cells treated with miR-199a inhibitor (Figures 1K,L). These observations demonstrate that miR-199a decreases macrophage-mediated killing of *M. bovis*.

### miR-199a directly targets TBK1

**Figure 2:**
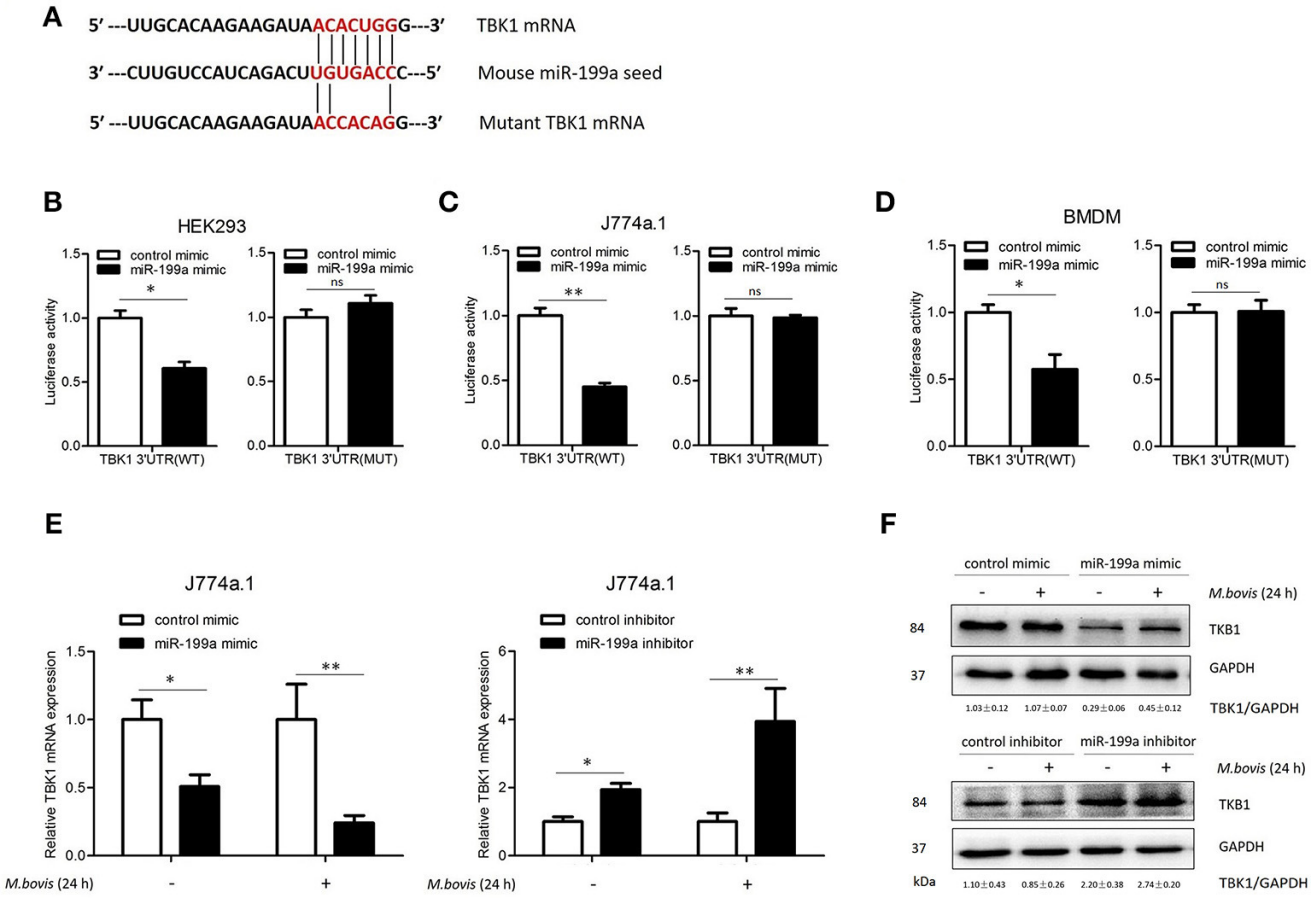
miR-199a inhibits the expression of TANK-binding kinase 1 (TBK1) by targeted TBK1 3′UTR. **(A)** Bioinformatics software identification of the miR-199a target gene HEK293 cells **(B)**, J774a.1 cells **(C)**, and mouse bone marrow monocyte-derived macrophages (BMDMs) **(D)** were cotransfected with pmirGLO vector carrying TBK1 WT or mutant constructs (MUT), along with a miR-199a mimic, and a luciferase assay was performed. **(E)** J774a.1 cells were treated with miR-199a mimic or inhibitor and then either uninfected or infected with *Mycobacterium bovis* (*M. bovis*) at a MOI of 10 for 24 h. TBK1 expression was determined by Quantitative Real-Time PCR. **(F)** Western blots of TBK1 protein expression levels. Quantitative analysis of TBK1 normalized to GAPDH is shown below each blot. Data is presented as mean ± *SD* in three independent experiments (*P < 0.05; **P < 0.01).

To screen specific mRNA targets with miR-199a-binding sites, we used a bioinformatics analysis from TargetScan. The list of putative miR-199a targets (Supplementary Table S2) showed that miR-199a is likely to impact a number of processes and pathways that are of importance in innate immunity and autophagy. TBK1 mRNA was selected as a potential target for miR-199a through interaction with complementary sequences, from 44 to 50 bp, at the 3′ UTR of TBK1 (ENST00000331710.5 3′ UTR length: 686 bp, http://www.targetscan.org) (Figure 2A). The 3′ UTR of TBK1 was cloned into a luciferase reporter construct (pmirGLO) and reporter assays were performed in HEK293, J774a.1 and BMDM cells. Using 3′ UTR luciferase reporter assays, we demonstrated miR-199a-dependent suppression of luciferase activity in HEK293, J774a.1 and BMDM cells co-transfected with pmirGLO-TBK1 WT 3′ UTR and miR-199a mimic (Figures 2B–D). Additionally, co-transfection of miR-199a mimic with pmirGLO-TBK1 MUT 3′ UTR did not changed luciferase activity, confirming that TBK1 was a putative target of miR-199a. To verify whether miR-199a targeted TBK1 in uninfected and *M. bovis-*infected cells, we examined TBK1 expression in J774a.1 cells transfected with miR-199a control, miR-199a mimic, or miR-199a inhibitor. As expected, miR-199a reduced the amount of TBK1 while inhibition of miR-199a increased TBK1 expression in cells at both mRNA and protein levels (Figure 2E,F and full WB blot in Supplementary Figure S2).

### miR-199a suppresses autophagosomes maturation by targeting TBK1

**Figure 3:**
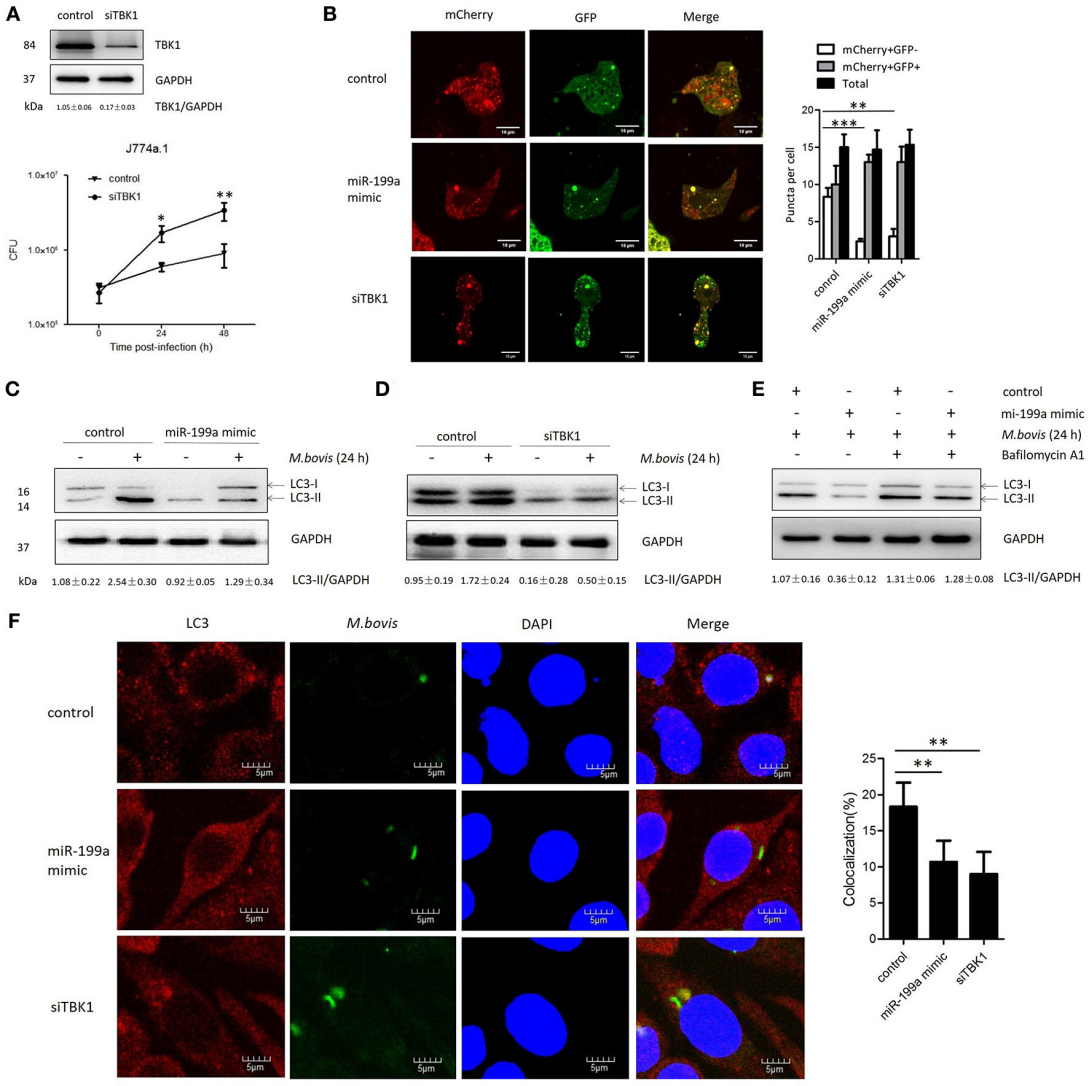
miR-199a suppresses autophagosomes maturation by target TANK-binding kinase 1 (TBK1).**(A)** Interference efficiency of TBK1 by small interference RNA (siTBK1). Universal scrambled RNA was used as a control for siTBK1 or miR-199a mimic. TBK1 protein level was analyzed using western blotting in J774a.1 cells transfected with control or siTBK1. J774a.1 cells were pretreated with control or siTBK1 and infected with *Mycobacterium bovis* (*M. bovis*). *M. bovis* survival was analyzed by colony-forming units (CFU). **(B)** J774a.1 cells expressing were infected with Ad-mCherry-GFP-LC3 for 24 h, then treated with control, miR-199a or siTBK1 for another 24 h and then infected with *M. bovis* at a MOI of 10 for 24 h. mCherry^+^GFP^+^, early autophagic organelles; mCherry^+^GFP^−^, late autophagic organelles. Images indicate merged green and red channels. 20 cells per group were counted for the relative fluorescence intensity. Image J software was used for the analysis. **(C,D)** Effect of miR-199a on LC3-II protein levels and degradation during autophagy. J774a.1 cells were pretreated with miR-199a mimic or siTBK1 for 24 h and then infected with *M. bovis* at a MOI of 10 for 24 h or left uninfected. The LC3-II /GAPDH ratios are shown below the blots. **(E)** Effect of miR-199a on LC3-II protein levels in the presence or absence of 100 nM bafilomycin A1 (24 h). Samples were then infected with *M. bovis* or left uninfected. The LC3-II /GAPDH ratios are shown below the blots. **(F)** Immunofluorescence staining of LC3 (red) of J774a.1 cells infected with Alexa 488-labeled *M. bovis* (green) at a MOI of 10 for 24 h (left). The proportion of LC3-positive mycobacteria is shown (right). Cell nuclei were dyed with DAPI (blue). One hundred cells were used to calculate the colocalization proportion. Image J software was used for the analysis. Data is presented as mean ± *SD* in three independent experiments (*P < 0.05; **P < 0.01; ***P < 0.001).

TBK1 is an important modulator for autophagy and mediates the maturation of autophagosomes into lytic bactericidal organelles (Pilli et al., 2012). We found that silencing of TBK1 led to a deficit in elimination of *M. bovis* (Figure 3A). We observed that TBK1 is a direct target of miR-199a. Therefore, we speculated that miR-199a might affect autophagosome maturation during *M. bovis* infection. We found that miR-199a mimic or siTBK1 inhibit autophagosomes maturation (autophagy flux) into autolysosomes. This was showed by the recombinant mCherry-GFP-LC3B adenovirus, which identifies matured autolysosomal organelles as mCherry^+^GFP^−^ and early autophagic organelles as mCherry^+^GFP^+^ because intracellular endosomal acidification would lead to GFP (but not mCherry) fluorescence quenching (Pilli et al., 2012). Consistent with our hypothesis, miR-199a mimic or siTBK1 reduced mCherry^+^GFP^−^ accumulation, and increased the number of mCherry^+^GFP^+^ (Figure 3B). The role of miR-199a in autophagosomal maturation was verified by LC3 western blotting. Cells transfected with miR-199a mimic or siTBK1 showed a decrease in LC3-II accumulation in both infected and un-infected cells with *M. bovis* (Figures 3C,D). To determine whether miR-199a regulates autophagosome formation or autophagy flux, bafilomycin A1, a known inhibitor of the latter stages of autophagy, was used in our experiments. Interestingly, the effect of miR-199a on autophagy is less obvious (Figure 3E) and consequently our findings demonstrate that miR-199a inhibited not only autophagosome formation but also autophagy flux. Further, we evaluate the effect of miR-199a on the colocalization of LC3 with *M. bovis* in J774a.1 cells. We observed a mark reduction in the colocalization of LC3 with *M. bovis* following overexpression of miR-199a or inhibition of TBK1 (Figure 3F). The above results demonstrate that, by targeting TBK1 mRNA, miR-199a has an inhibitory effect on *M. bovis*-induced autophagy.

### Transmission electron microscopy (TEM) confirms repression of autophagy by miR-199a

**Figure 4:**
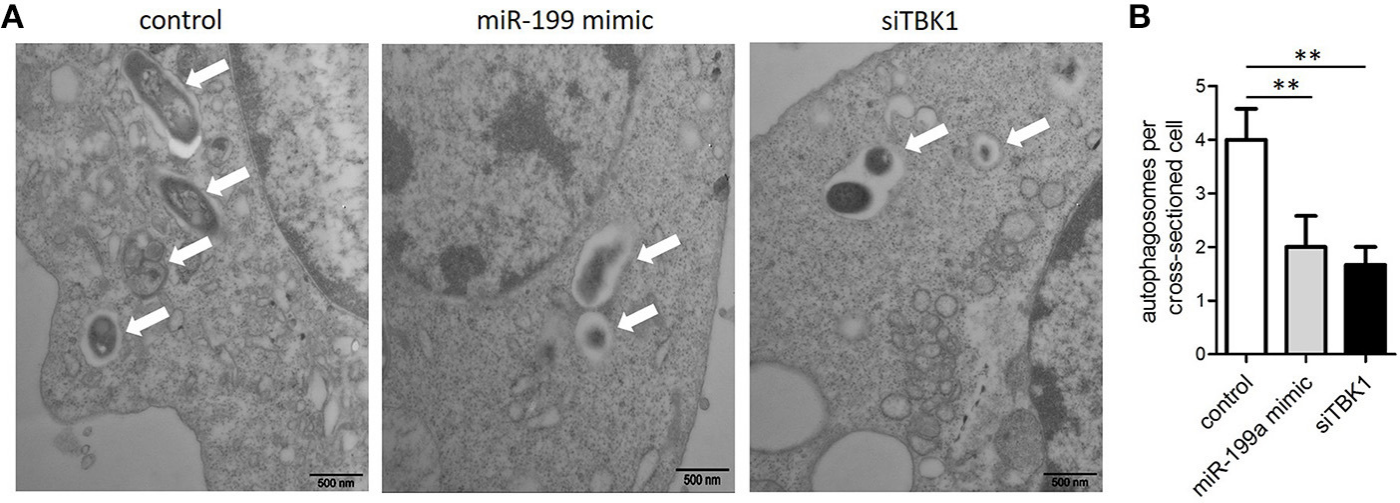
The inhibitory effect on autophagy by miR-199a was confirmed by Transmission Electron Microscopy (TEM). J774a.1 cells were transfected with control, miR-199a mimic or siTBK1 for 24 h, and then infected with *M. bovis* at an MOI of 10 for 24 h. **(A)** Close-up images (× 4,0000 magnification) of cytoplasmic regions containing autophagosomes (denoted by white arrowheads). **(B)** The number of autophagosomes per cross-sectioned cell was counted (20 cells per group counted by TEM). Data is presented as mean ± *SD* in three independent experiments (**P < 0.01).

In order to confirm that miR-199a inhibits autophagy in macrophages, autophagosomes in cellular cross-sections were imaged and quantified by TEM. Representative TEM images are shown in Figure 4A. Transfection with miR-199a mimic reduced the number of autophagosomes per cellular cross-section compared to transfection with control in *M. bovis*-infected J774a.1 cells. Moreover, there was a significant additional decrease in the number of autophagosomes per cellular cross-section upon transfection with siTBK1 (Figure 4B), confirming our immunofluorescence and western blotting analysis results (Figure 3).

### miR-199a inhibits activation of TBK1-IRF3-IFN-β axis in *M. bovis*-infected cells

**Figure 5:**
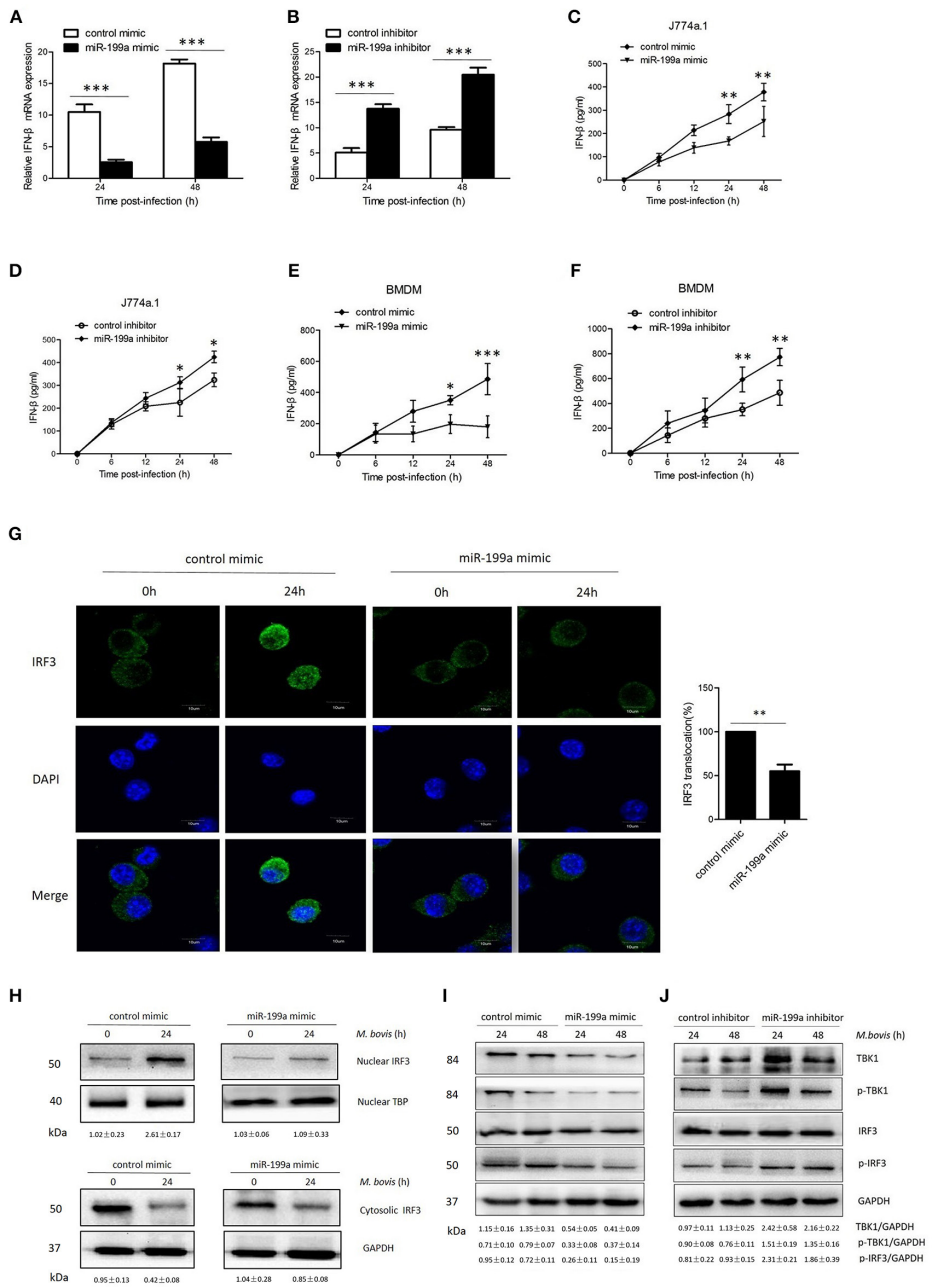
miR-199a impairs interferon-β (IFN-β) production by inhibiting TANK-binding kinase 1 (TBK1)-dependent pathway. J774a.1 cells were treated with miR-199a mimic or inhibitor for 24 h, and then infected with *Mycobacterium bovis* (*M. bovis*) at a MOI of 10 for the indicated time. mRNA levels of IFN-β **(A,B)** were determined by Quantitative Real-Time PCR. J774a.1 cells and mouse bone marrow monocyte-derived macrophages (BMDMs) were treated with miR-199a mimic or inhibitor for 24 h, followed by *M. bovis* infection at a MOI of 10 for the indicated time. IFN-β production **(C–F)** was measured by ELISA assay. **(G)** Immunofluorescence staining of IRF3 (green) of J774a.1 cells infected with *M. bovis* at a MOI of 10 for 0 or 24 h. The proportion of nuclear IRF3 is shown (right). Cell nuclei were dyed with DAPI (blue). Approximately 20 cells were used to calculate the IRF3 nuclear translocation proportion. Image J software was used for the analysis. **(H)** J774A.1 cells were infected with *M. bovis* at a MOI of 10 for 0 or 24 h. Nuclear and cytosolic extracts were examined by immunoblotting with anti-IRF3 antibody at different infection times (4, 24 h). These membranes were stripped and immunoblotted with anti-β-actin and anti-TATA binding protein (TBP) antibodies to examine the purity of nuclear (right) and cytoplasmic (left) lysates. The IRF3/β-actin and IRF3/β-actin ratios are shown below. **(I, J)** J774a.1 cells were treated with control, miR-199a mimic or inhibitor and then infected with *M. bovis* at a MOI of 10 for 24 or 48 h. Quantitative analysis of the TBK1, phosphorylated TBK1 (p-TBK1), IRF3 and phosphorylated IRF3 (p-IRF3) bands normalized to GAPDH is shown. Data is presented as mean ± *SD* in three independent experiments (*P < 0.05; **P < 0.01; ***P < 0.001).

As TBK1 also plays an important role in the induction of type I interferons, we therefore investigated whether miR-199a had an effect on IFN-β. J774a.1 cells and BMDMs were transfected with control or miR-199a mimic followed by *M. bovis* challenge, and the transcription of IFN-β gene and the production of IFN-β were detected by real-time PCR and ELISA, respectively. Overexpression of miR-199a mimic reduced IFN-β mRNA expression (Figure 5A) and IFN-β production (Figures 5C,E) in *M. bovis*-challenged J774a.1 cells and BMDMs. Consistently, when endogenous miR-199a was inhibited, both IFN-β mRNA and IFN-β production increased in J774a.1 cells and BMDMs (Figures 5B,D,F).

To determine the mechanism by which miR-199a suppressed IFN-β production, we examined the IRF3 signaling pathways, which is critical for the host immune response. Resting IRF3 distributes in the cytoplasm, while activated IRF3 form dimers and translocate into nucleus to initiate the transcription of IFN-β gene (Mohr and Sonenberg, 2012; Ysebrant de Lendonck et al., 2014). We observed attenuated nuclear translocation of IRF3 in miR-199a-overexpressed cells using confocal microscopy (Figure 5G). We then extracted proteins from nuclei and cytoplasm, and examined the protein levels of IRF3 to determine the activation of the pathway by western blotting. The results confirmed that miR-199a inhibited nuclear accumulation of IRF3, suggesting inhibition of IRF3 activation (Figure 5H). The above results indicate that miR-199a impairs *M. bovis*-induced IFN-β production in macrophages by inhibiting IRF3 translocation to the nucleus.

To examine whether miR-199a can affect the TBK1-dependent pathway, we used western blotting to monitor the protein expression of TBK1, p-TBK1, IRF3, and p-IRF3 in J774a.1 cells a t 24 or 48 h post-infection. We observed less TBK1, p-TBK-1, and p-IRF3 expression in cells in which miR-199a was overexpressed (Figure 5I). On the contrary, downregulation of miR-199a showed opposite results (Figure 5J) demonstrating that miR-199a inhibits the TBK1-associated signaling response during *M. bovis* infection.

### TBK1 promotes antimycobacterial responses through a mechanism involving activation of autophagy

**Figure 6:**
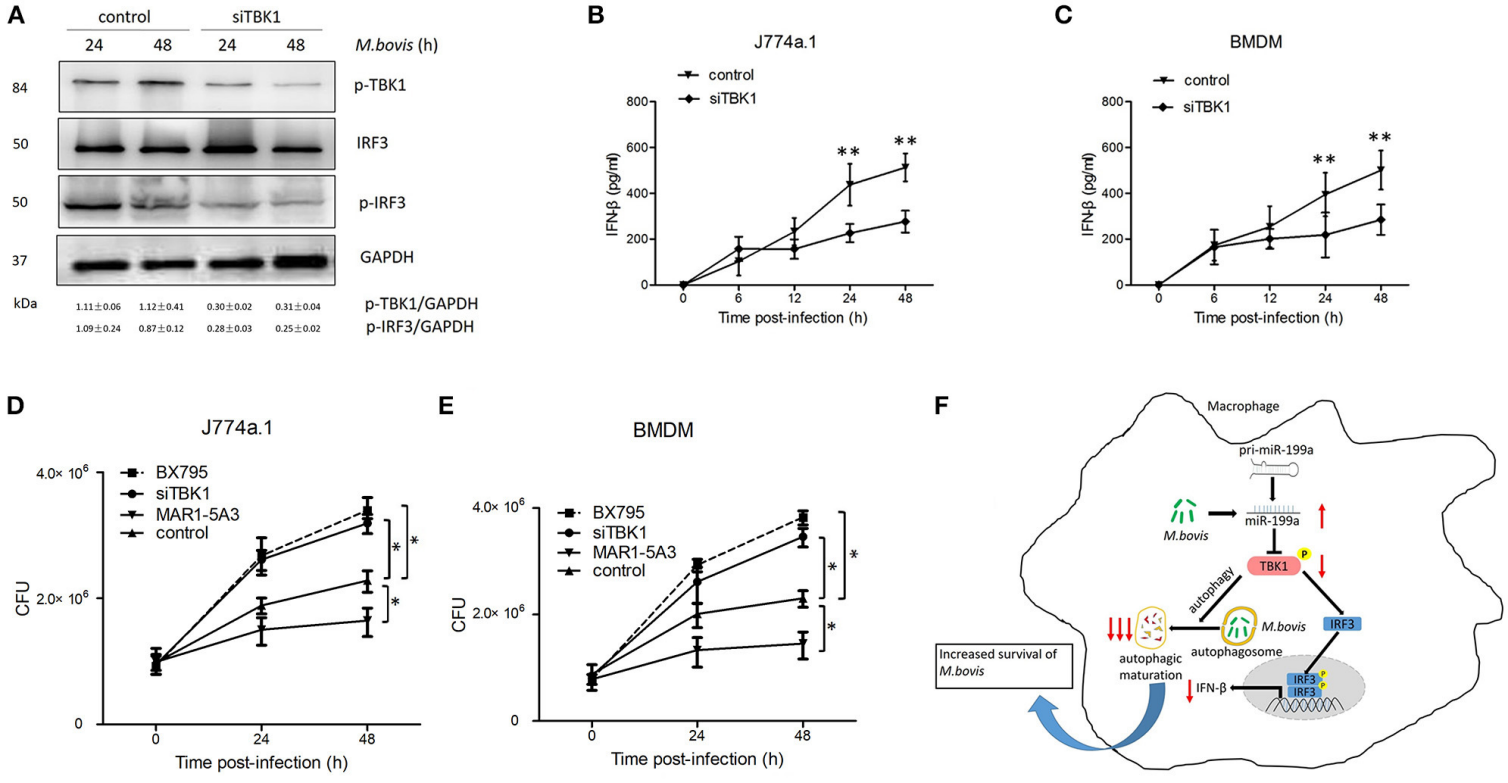
TANK-binding kinase 1 (TBK1) attenuates *Mycobacterium bovis* (*M. bovis*) survival *in vitro* by control of autophagy and the production of interferon-β (IFN-β). **(A)** J774a.1 cells were treated with control or siTBK1 for 24 h and then infected with *Mycobacterium bovis* (*M. bovis*) at a multiplicity of infection (MOI) of 10 for 24 or 48 h. Quantitative analysis of the phosphorylated TBK1 (p-TBK1), IRF3 and phosphorylated IRF3 (p-IRF3) bands normalized to GAPDH is shown. **(B)** J774a.1 cells and **(C)** mouse bone marrow monocyte-derived macrophages (BMDMs) were treated with control or siTBK1 for 24 h, followed by *M. bovis* infection at a MOI of 10 for the indicated time. Production of IFN-β was measured by ELISA assay. **(D)** J774a.1 cells and **(E)** mouse bone marrow monocyte-derived macrophages (BMDMs) were treated with control, siTBK1 for 24 h, BX795 (5 μM, 2 h) and IFNAR1-blocking monoclonal antibody MAR1-5A3 (1 μg/ml, 2 h), and then infected with *Mycobacterium bovis* (*M. bovis*) at a multiplicity of infection (MOI) of 10 for 24 or 48 h. *M. bovis* survival was analyzed by colony-forming units (CFU). **(F)** Model depicting the cascade of events following *M. bovis* infection involving sequential upregulation of miR-199a; downregulation of TBK1; suppression of autophagosomes maturation; downregulation of IFN- β and increased survival of *M. bovis*. The red arrows show an inhibition of TBK1 by miR-199a seems high importance for the abrogation of autophagy than down regulation of IFN-β via TBK1/IRF3 axis. Data is presented as mean ± *SD* in three independent experiments (*P < 0.05; **P < 0.01).

Macrophages from mice lacking TBK1 suppressed the production of IFN-β and modulated normal T cell function, as well as ablated IRF3 DNA-binding activity (Marchlik et al., 2010). Thus, we asked whether TBK1 is required for the survival and growth of *M. bovis* via induction of IFN-β in infected macrophages. Therefore, we silenced TBK1 expression in macrophages to investigate the role of IFN-β in *M. bovis* survival. We found that TBK1 silencing significantly decreased TBK1, p-TBK-1, and p-IRF3 expression in *M. bovis* infected J774a.1 cells at both 24 and 48 h post-infection (Figure 6A). In addition, we observed that TBK1 silencing significantly reduced the production of IFN-β in J774a.1 and BMDM macrophages after 24 and 48 h post-infection with *M. bovis* (Figures 6B,C). TBK1 also play an important role in the regulation of autophagy during *M. tuberculosis* infection (Pilli et al., 2012). In order to determine whether TBK1 contributes to *M. bovis* survival in macrophages by promoting autophagy, CFU was conducted to determine the bacterial load in different treatment groups. Treatment with 5 μM BX795 (a pharmacological inhibitor of TBK1, also blocker of IFN-β) and siTBK1 increased the bacterial load of intracellular *M. bovis* in both J774a.1 and BMDM macrophages after 24 and 48 h of infection. Furthermore, blocking of type I IFN receptor subunit IFNα/β receptor 1 (IFNAR1) by using monoclonal antibodies MAR1-5A3 (Biolegend) resulted in a decreased bacterial load of *M. bovis* in macrophages (Figures 6D,E). These findings shows that TBK1 is necessary for the regulation of autophagy and the elimination of *M. bovis* from infected macrophages and dominate IFN-β role in the survival of *M. bovis*. Taken together, our findings suggest that *M. bovis* persistently survive in infected macrophages by upregulating the expression of miR-199a. While miR-199a negatively regulates autophagic flux and innate immune responses via targeting TBK1 in *M. bovis* infected macrophages. (Figure 6F).

## Discussion

MiRNAs control a variety of biological processes and cell signaling pathways associated with homeostasis, development and diseases. They are also key regulators of inflammation and innate immunity (Junger, 2011; O'Connell et al., 2012). miR-29 regulates NK cell function and Th1-type responses to intracellular pathogens by targeting IFN-γ mRNA (Ma et al., 2011). BCG upregulates the expression of miR-20a leading to the downregulation of its target protein ATG7 and ATG16L1, a process that could prevent autophagy (Guo et al., 2016). In addition, miR-27a activate macrophage responses to control intracellular survival of *Mycobacterium avium* subspecies *paratuberculosis* by targeting TAB2 and IL-10 mRNA (Hussain et al., 2018). Early study reported that *M. bovis* infection upregulate the expression of miR-146a and miR-146b in alveolar macrophages. Furthermore, it is demonstrated that miR-146 target IL-1 receptor associated kinase 1 (IRAK1), leads to inhibition of innate immune signaling pathways (Vegh et al., 2014). It suggests that intracellular mycobacterium including *M. bovis* adopted strategies to manipulate host miRNAs regulation for its own benefit and survival. In the present study, we focused on the role of miR-199a on innate immune responses in murine macrophages infected with *M. bovis*. Previous studies reported that miR-199a reduces interleukin-8 (IL-8) secreation through inhibition of IKKβ/NF-κB pathway in endometrial stromal cells (Cheung et al., 2011; Dai et al., 2012). In addition, miR-199a impaired cardiomyocyte autophagy in a cell-autonomous manner by targeting glycogen synthase kinase 3β (GSK3β)/mammalian target of rapamycin (mTOR) complex signaling pathway in cardiac-specific miR-199a transgenic mice (Li et al., 2017). Similarly, we found substantial differences in autophagic responses and production of IFN-β between *M. bovis* infected and uninfected macrophages in response to miR-199a treatment. Consistent with previous studies with *M tuberculosis*, we found that miR-199a expression decreased in early *M. bovis*-infected macrophages for 24 h, but after extending the analysis up to 48 h, it markedly increased in a time- and dose-dependent manner. In line with this notion, no significant difference was found in the expression of miR-199a in liver samples of *M. bovis*-infected and uninfected mice, while an increase expression was observed in *M. bovis*-infected spleens and lungs. Using CFU assays, we found that miR-199a attenuates the clearance of mycobacteria in macrophages. However, the potential role of miR-199a on immune responses during *M. bovis* infection remains unclear, so we hypothesized that miR-199a may be a regulator that modulates early events associated with *M. bovis* infection.

Innate immunity is a conserved host mechanism in multicellular organisms and the first line of defense against pathogens. Several groups have recently proposed a mechanism for IFN-β induction by *M. tuberculosis*. When *M. tuberculosis* gains access to the host cytosol (Stanley et al., 2007), STING is triggered by bacterial cyclic dinucleotides (Dey et al., 2015) or through DNA binding to cyclic GMP-AMP synthase (cGAS) in the cytosol. Activated STING then combines and activates the kinase TBK1, which induces the activation of the transcription factor IRF3. The transcription factor IRF3 translocates into the nucleus to initiate IFNB1 gene transcription. TBK1, as a key node kinase, controls dsDNA-mediated IRF3 signaling pathways (Collins et al., 2015; Wassermann et al., 2015; Watson et al., 2015). Besides, TBK1 involves the degradation of invading bacteria, through ubiquitin-mediated scavenging machinery, activating autophagy to kill invasive bacteria (Wild et al., 2011). TBK1 also plays an important role in engaging selective autophagy pathways for efficient clearance of injured mitochondria (Wong and Holzbaur, 2014). Our results indicate that TBK1 is a putative target of miR-199a. Using luciferase reporter assays, we demonstrated that miR-199a decreased the relative luciferase activity in macrophages expressing the TBK1-WT 3′ UTR reporter, but did not suppress luciferase activity in macrophages expressing the TBK1-MUT 3′ UTR reporter. Moreover, miR-199a upregulation reduced the expression of TBK1 in macrophages, whereas transfection of cells with miR-199a inhibitor led to TBK1 accumulation in macrophages.

Autophagy, which is affected by miRNAs, plays a key role in cleaning *M. tuberculosis* infection. For instance, miR-125a expression increased in mouse macrophages upon *M. tuberculosis* infection. This is because miR-125a controls the innate host defense by inhibiting cellular autophagy against *M. tuberculosis* by targeting UV radiation resistance- associated gene (Kim et al., 2015). It is reported that, miR-155, facilitates cellular autophagy for the removal of mycobacteria by targeting Ras homolog enriched in brain (Wang et al., 2013). Another study has reported that BCG upregulates the expression of miR-20a leading to the downregulation of its target protein ATG7 and ATG16L1, a process that could prevent autophagy (Guo et al., 2016). As mentioned above, miR-199a has the ability to inhibit cellular autophagy via mTOR activation (Li et al., 2017). Our study shows that miR-199a suppressed maturation into autolysosomes in macrophages via TBK1, providing new insights into mycobacteria-mediated autophagy. Quantification of autophagosomes per cellular cross-section revealed a significant reduction in cells transfected with miR-199a mimic or siTBK1, confirming our results of immunofluorescence analysis. TBK1 is required for production of type I interferon during *M. tuberculosis* infection (Stanley et al., 2003) but it is also necessary for selective targeting of *M. tuberculosis* to autophagy (Watson et al., 2012). Thurston et al., reported that recruitment of TBK1 to cytosol-invading bacteria is essential for anti-bacterial autophagy (Thurston et al., 2016). The function of miR-199a in relationship to the host defense against *M. bovis* is largely unknown. We found that the survival of *M. bovis* in infected macrophages was facilitated in the absence of TBK1, miR-199a overexpression, suggesting that miR-199a promoted the survival of *M. bovis* in macrophages by targeting TBK1.

Low concentrations of type I interferons is required for protection, especially early in *M. tuberculosis* infection. However, several studies have shown that higher levels of IFN-α and β are associated with minimum outcomes of infection with pathogenic mycobacteria (Teles et al., 2013). For example, mice lacking the IFN-α/β receptor have the ability to attenuate *M. tuberculosis* growth in spleens, illustrating that IFN-α/β is harmful to the body (Stanley et al., 2007). IFN-α/β promotes the pathogenesis during chronic *M. africanum* infection which showed that this infection may be mediated through in-efficient control of bacterial growth (Wiens and Ernst, 2016). Consistent with previous studies with *M tuberculosis*, we found that treatment with the monoclonal antibody MAR1-5A3 significantly inhibited the intracellular load of *M. bovis* in macrophages. While, treatment with miR-199a mimic, BX795 and siTBK1 significantly increased the intracellular load of *M. bovis* in macrophages. Our results suggest that, the decrease in autophagy caused by miR-199a inhibition of TBK1 seems to be dominant in promoting *M. bovis* survival over the opposing effect of reduced IFN-β production.

In conclusion, our study shows that miR-199a is upregulated by *M. bovis* infection, and inhibits the autophagic response and decreases the production of IFN-β in macrophages via targeting TBK1, conferring that *M. bovis* has the ability to evade immune clearance. Moreover, the inhibitory effect of miR-199a on TBK1-autophagy-mycobacterial control appears to be dominant over IFN-β signaling for the survival of *M. bovis* (Figure 6F). This demonstrates a key role of miR-199a in the regulation of autophagy and innate immune adjustment, providing a better understanding of the mechanism by which *M. bovis* escape from the immune clearance and promote pathogenesis.

## Author contributions

JW wrote the manuscript. DZ and XZ designed experiments. JW, RY, TH, YL, and QL performed experiments. JY, YS, XS, NW, and LX assisted to complete experiments. SS rewrote and edited the manuscript.

### Conflict of interest statement

The authors declare that the research was conducted in the absence of any commercial or financial relationships that could be construed as a potential conflict of interest.

## Supplementary material

Supplementary Figure S1 The sequence of miR-199a and miR-199b similar target TBK1 at 3′UTR from target scan.

Supplementary Figure S2 Full blot images of western blot results with expected weights along with a protein ladder. **(A)** a full blot image of TBK1 protein already shown in Figure 2F (B) a full blot image of p-IRF3 protein already shown in Figure 5I (C) a full blot image of p-IRF3 protein already shown in Figure 6A.

Supplementary Table S1 Gene sequences of miR-199a and TBK1 used in cells transfection experiments.

Supplementary Table S2 Predicted targets of miR-199a from target scan.
