# Supplementary figures and images for: MicroRNA-199a Inhibits Cellular Autophagy and Downregulates IFN-β Expression by Targeting TBK1 in Mycobacterium bovis Infected Cells
Source: Front Cell Infect Microbiol. 2018 Jul 10;8:238. doi: 10.3389/fcimb.2018.00238 (PMC6048223; doi:10.3389/fcimb.2018.00238)

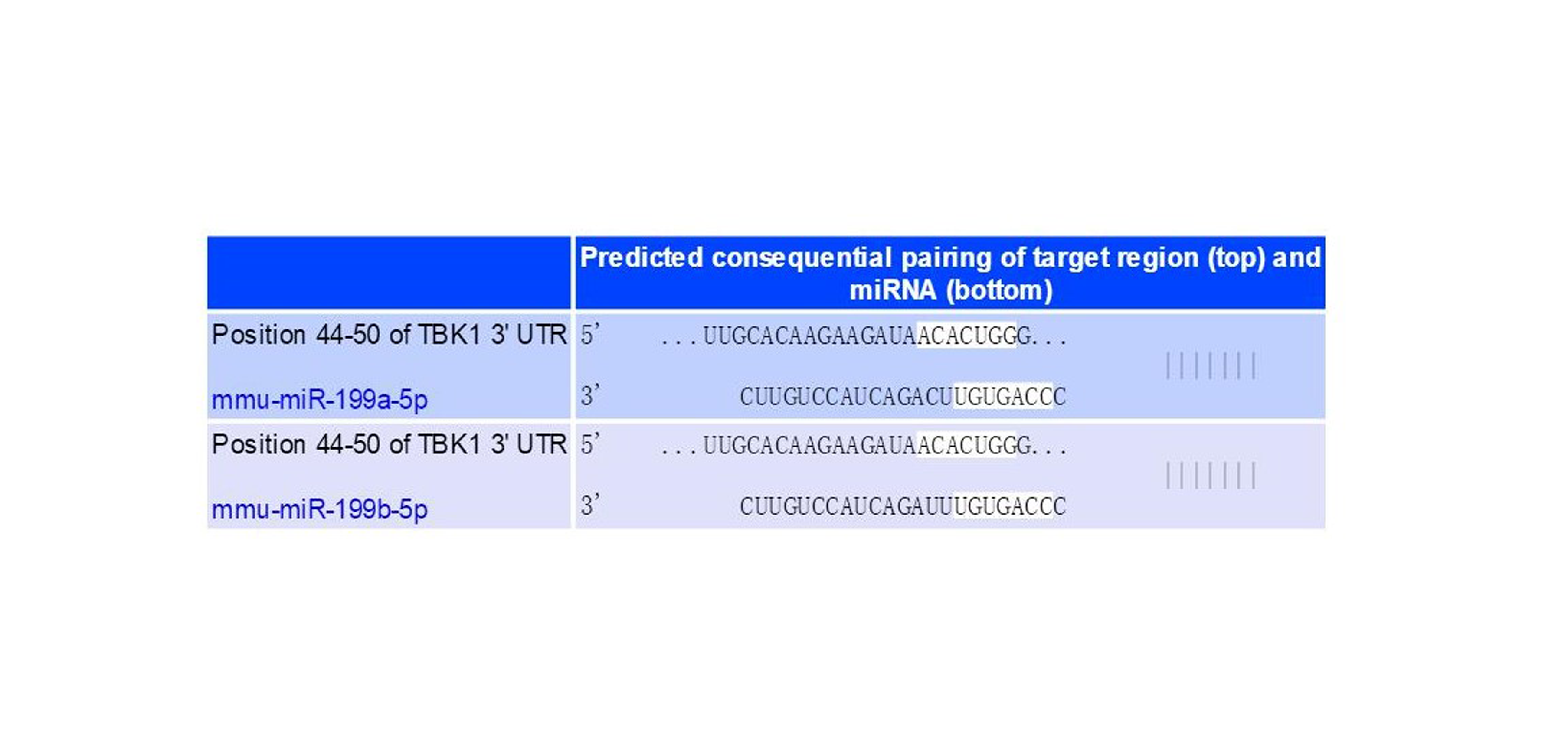

Supplement: Supplementary Figure S1 — The sequence of miR-199a and miR-199b similar target TBK1 at 3′UTR from target scan. [file Image_1.TIF]

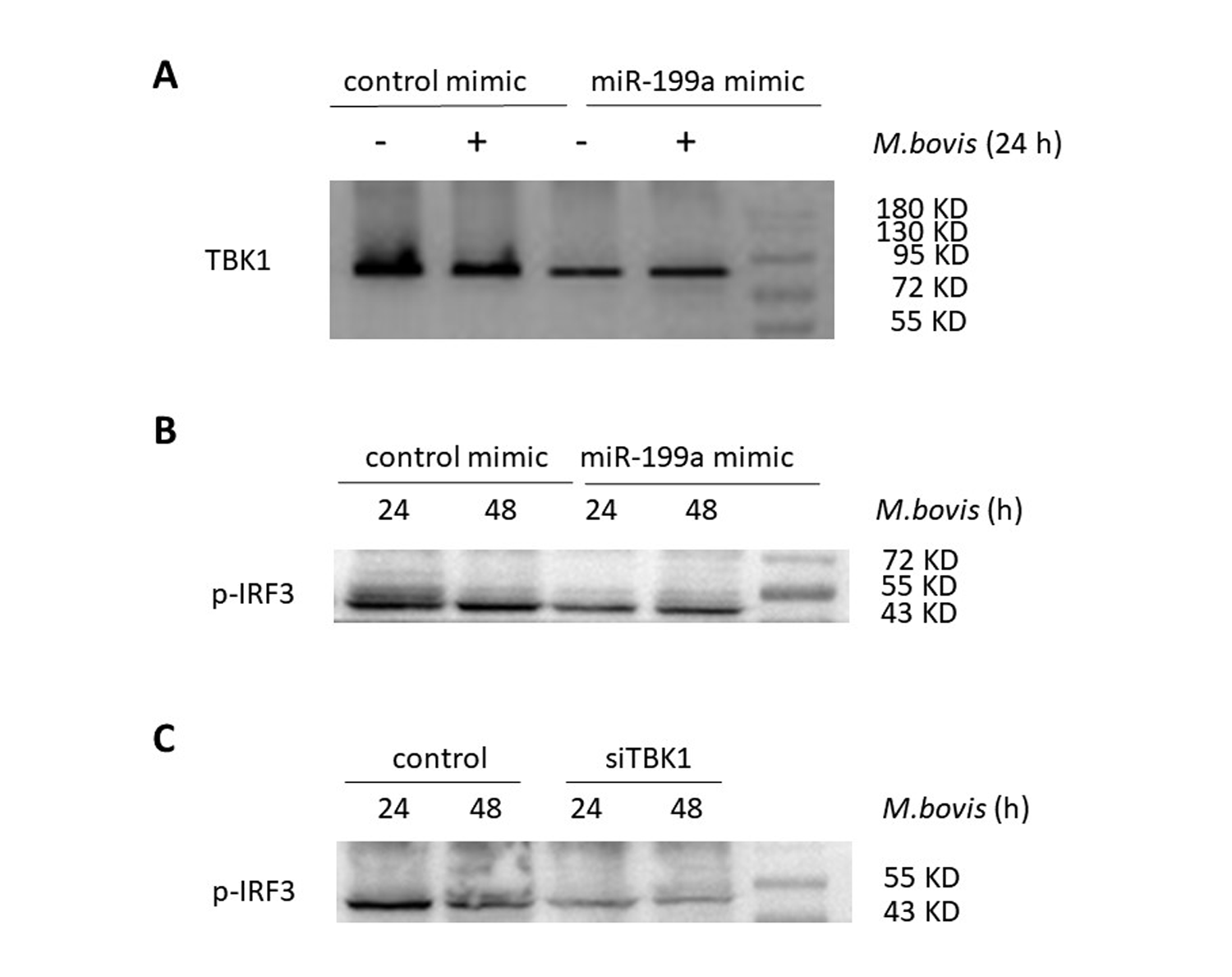

Supplement: Supplementary Figure S2 — Full blot images of western blot results with expected weights along with a protein ladder. (A) a full blot image of TBK1 protein already shown in Figure 2F (B) a full blot image of p-IRF3 protein already shown in Figure 5I (C) a full blot image of p-IRF3 protein already shown in Figure 6A. [file Image_2.TIF]
