# Supplementary material for: MicroRNA-199a Inhibits Cellular Autophagy and Downregulates IFN-β Expression by Targeting TBK1 in Mycobacterium bovis Infected Cells
Source: Front Cell Infect Microbiol. 2018 Jul 10;8:238. doi: 10.3389/fcimb.2018.00238 (PMC6048223; doi:10.3389/fcimb.2018.00238)
Supplement: Supplementary Table S1 — Gene sequences of miR-199a and TBK1 used in cells transfection experiments. [file Data_Sheet_1.docx]

**Supplementary Tables**

**Table S1.** Gene sequences of miR-199a and TBK1 used in cells transfection experiments

| Gene name | Sequence (sense, antisense) |
| --- | --- |
| control mimic(control) | UUCUCCGAACGUGUCACGUTT; ACGUGACACGUUCGGAGAATT |
| miR-199a mimic | CCCAGUGUUCAGACUACCUGUUC; ACAGGUAGUCUGAACACUGGGUU |
| control inhibitor | CAGUACUUUUGUGUAGUACAA |
| miR-199a inhibitor | GAACAGGUAGUCUGAACACUGGG |
| siTBK1 | GAAGCCGUCUGGUGCAAUA; UGACGGCGCAUAAGAUUUA; CUACGAAGGACGACGCUUA; GUAUGAAGCGUUUAAAGAU |

**Table S2.** Predicted targets of miR-199a from target scan

| Target gene | Representative transcript | Gene name |
| --- | --- | --- |
| TBK1 | ENST00000331710.5 | TANK-binding kinase 1 |
| TMEM173 | ENST00000330794.4 | transmembrane protein 173 |
| TRAF3 | ENST00000560371.1 | TNF receptor-associated factor 3 |
| IKBKB | ENST00000379708.3 | inhibitor of kappa light polypeptide gene enhancer in B-cells, kinase beta |
| GSK3B | ENST00000264235.8 | glycogen synthase kinase 3 beta |
| ULK1 | ENST00000321867.4 | unc-51 like autophagy activating kinase 1 |
| ATG2B | ENST00000359933.4 | autophagy related 2B |
| ATG4B | ENST00000404914.3 | autophagy related 4B, cysteine peptidase |
| ATG5 | ENST00000360666.4 | autophagy related 5 |
| ATG12 | ENST00000500945.2 | autophagy related 12 |
| ATG13 | ENST00000359513.4 | autophagy related 13 |
| ATG14 | ENST00000247178.5 | autophagy related 14 |
| ATG16L1 | ENST00000392017.4 | autophagy related 16-like 1 (S. cerevisiae) |
| RAB7A | ENST00000265062.3 | RAB7A, member RAS oncogene family |
| BAX | ENST00000391871.3 | BCL2-associated X protein |
